# Infections in Hospitalised Patients with Multiple Myeloma: Main Characteristics and Risk Factors

**DOI:** 10.4274/tjh.2013.0173

**Published:** 2015-08-01

**Authors:** Toni Valković, Vedrana Gačić, Jelena Ivandić, Božo Petrov, Renata Dobrila-Dintinjana, Elizabeta Dadić-Hero, Antica Načinović-Duletić

**Affiliations:** 1 University Hospital Centre Rijeka, Clinic of Haematology, Rijeka, Croatia; 2 University Hospital Mostar, Clinic of Haematology, Mostar, Bosnia and Herzegovina; 3 University Hospital Centre Rijeka, Clinic of Gynaecology and Obstetrics, Rijeka, Croatia; 4 University Hospital Mostar, Clinic of Psychiatry, Mostar, Bosnia and Herzegovina; 5 University Hospital Centre Rijeka, Clinic of Oncology and Radiotherapy, Rijeka, Croatia; 6 Community Primary Health Centre, Primorsko-Goranska County, Rijeka, Croatia

**Keywords:** Multiple myeloma, infections, risk factors, therapy

## Abstract

**Objective:**

Multiple myeloma is a common haematological malignancy and immune dysfunction is the hallmark of the disease. It leads to an increased infection risk, which is still a major cause of mortality. The infection spectrum and characteristics have evolved with the introduction of novel agents. An understanding of risk factors that increase susceptibility to infections is critical in fighting them. This retrospective investigation aimed to establish the incidence and main characteristics of infections in non-transplanted hospitalised myeloma patients in our department over a 3-year period, as well as factors associated with infections.

**Materials and Methods:**

A total of 240 hospitalised patients with multiple myeloma (120 males and 120 females; average age: 69 years, range: 41-89 years) who were diagnosed or treated in our department from January 2008 to December 2010 were included in this study and their data were retrospectively analysed.

**Results:**

Infections were identified in 17.9% of hospitalised patients. The most common pathogen found was Pseudomonas aeruginosa. The frequency of gram-positive and gram-negative pathogens was similar. In 37.2% of cases, the agent was not isolated. The most common sites of infections were the urinary system and the blood (septicemia). The frequency of infection increased with duration of disease and the rate of reinfection was 41.9%. The patients treated with bortezomib had the highest infection occurrence. Fatal outcome occurred in 9.3% of cases.

**Conclusion:**

The factors associated with infections in this investigation were female sex, 3B clinical stage of disease, increased serum creatinine and ferritin levels, neutropenia, poor general condition, and presence of catheters. Myeloma patients with one or more of these mentioned risk factors should be monitored with particular care in order to decrease the incidence and severity of infective complications.

## Abstract (tr)

**Amaç:**

Multipl miyelom, sık görülen bir hematolojik malignite olup, bağışıklık sistemi disfonksiyonu bu hastalığın en belirgin özelliğidir. Bu hastalığın sebep olduğu ileri düzeyde enfeksiyon riski hala başlıca ölüm sebebidir. Enfeksiyonun spektrumu ve özellikleri, yeni geliştirilen ajanlarla birlikte değişiklik göstermektedir. Enfeksiyonlara hassasiyeti arttıran risk faktörlerinin anlaşılması, bu enfeksiyonlara karşı savaşmak için son derece önemlidir. Bu retrospektif çalışma neticesinde 3 yıldır departmanımızda yatırılarak tedavi gören ve transplantasyon yapılmamış miyelom hastalarımızda görülen enfeksiyonların temel özelliklerinin saptanması amaçlanmıştır.

**Gereç ve Yöntemler:**

Ocak 2008 ile Aralık 2010 tarihleri arasında departmanımızda tanısı konmuş ve tedavi görmüş toplam 240 multipl miyelom hastası (120 erkek ve 120 kadın; ortalama yaş: 69, yaş aralığı: 41-89) bu çalışmaya dahil edilmiştir ve bu hastalardan elde edilen veriler retrospektif olarak incelenmiştir.

**Bulgular:**

Hastanemizde yatan hastaların %17,9’unda enfeksiyona rastlandı. En sık görülen patojenin Pseudomonas aeruginosa olduğu saptandı. Gram pozitif ve gram negatif patojenlerin sıklığı aynıydı. Olguların %37,2’sinde ajan izole edilemedi. Enfeksiyon kaynağının sıklıkla üriner sistem ve kan olduğu görüldü. Hastalık süresince enfeksiyon oranının arttığı ve reenfeksiyon sıklığının % 41,9 olduğu tespit edildi. En sık enfeksiyon bortezomib ile tedavi edilen hastalarda görüldü. Olguların %9,3’ü ölümle sonuçlandı.

**Sonuç:**

Bu çalışmada enfeksiyonlarla ilişkilendirilen faktörler kadın cinsiyet, ileri evre (III 3B) hastalık, artmış serum kreatinin ve ferritin düzeyi, nötropeni, genel durumun bozukluğu ve kateterlerin varlığıydı. Bahsi geçen bu faktörlerden bir ya da ikisine sahip miyelom hastalarda görülebilecek enfektif komplikasyon oranını ve şiddetini düşürmek amacıyla hastaların özenli ve dikkatli bir bakımla izlenmesi gerekmektedir.

## INTRODUCTION

Multiple myeloma (MM) is a common haematological malignancy, and immune dysfunction is one of the hallmarks of the disease. The disease-related immunological defects, as well as therapy-related immunosuppression, lead to the increased risk of infective complications, which are a major cause of mortality.

Malignant plasma cells produce monoclonal paraprotein, which is non-functional and crowds out the polyclonal immunoglobulins. Hypogammaglobulinaemia, which is related to suppression of CD19+ B lymphocytes, exists in a great majority of patients with the active disease [1]. Besides hypogammaglobulinaemia, numerous immunological abnormalities have been observed, such as several defects of T lymphocytes, dendritic cells, natural killer cells, and the complement cascade [2,3,4,5,6,7]. Quantitative and qualitative disorders of neutrophils are related to myelosuppressive therapy or bone marrow infiltration [8,9]. Furthermore, several other risk factors increase susceptibility to infections in MM, such as renal failure, smoking, using of various catheters, vertebral collapse, iron overload, or use of opiate drugs [10,11].

The spectrum and characteristics of infective complications in myeloma patients have evolved with the introduction of autologous and allogeneic hematopoietic stem cell transplant (HSCT) or different novel drugs, which together cause an increasing susceptibility to fungal and viral infections [12,13,14,15].

The present study has analysed the frequency and characteristics of infective complications, as well as factors related to risk for infections, in the myeloma patients treated at our institution over a 3-year period.

## MATERIALS AND METHODS

Retrospectively, using hospital medical documentation, 240 cases of hospitalised patients with MM (120 males and 120 females; average age of 69, range of 41-89 years) who were diagnosed or treated in our department from January 2008 to December 2010 were processed. Because the majority of patients were hospitalised more than once, the total number of our cases was larger than the number of patients included in this study (37 males and 35 females, respectively). Only patients who were not treated at the time of this study with high-dosage therapy and HSCT were included. The diagnosis was established according to International Myeloma Working Group criteria [16]. The great majority of patients in this study had IgG, IgA, or a light chain myeloma; however, 1 patient with IgD and 1 patient with nonsecretory myeloma were also included. Our patients were treated fairly uniformly. VAD (vincristine, doxorubicin, and dexamethasone) and MP (oral melphalan and prednisone) regimens were mostly used as the induction therapy (according to the patient’s age and eligibility for high-dose therapy), with thalidomide-based protocols as second-line therapy and bortezomib-based protocols as third- or next-line therapy. Monotherapy with dexamethasone was used as a front-line therapy, as well as for postinduction protocol in some patients.

The study was approved by the Medical School of Rijeka Ethics Committee.

The criteria for infection used in our study were increased body temperature above the normal range (37 °C) or isolation of a microbial agent in patients who also had concomitant clinical symptoms and/or humoral signs of infections (leukocytosis, neutrophilia, marked “left shift”, or increased C reactive protein in comparison with a baseline value).

The frequency and the basic characteristics of the identified infections, such as type of isolated microbial agents, site of infection, time of occurrence and outcome of infection, rate of re-infections, and type of antitumor therapy at the time of infection, were also determined. The factors that could influence the pathogenesis and incidence of infections, including sex, WHO/ECOG performance status [17], Durie-Salmon stage of disease [18], International Staging System (ISS) score [19], serum creatinine level, immuneparesis defined qualitatively (decreased serum concentration of any polyclonal immunoglobulin class), neutropenia (defined as blood neutrophil count of ≤2x109/L), serum ferritin level, and presence of any catheters, were compared and statistically analysed in cases with and without infections [17,18,19]. All these parameters according to the occurrence of infections are listed in Table 1.

### Statistical Analysis

Data were expressed as means ± standard deviations, with the sample size (n). Nominal and ordinal variables were analysed with the chi-square test. Normality of distribution of continuous variables was tested by the Kolmogorov-Smirnov test. For continuous variables with distribution significantly different from the norm, we used the median and interquartile range, and the differences between the groups for these variables were tested with the Kruskal-Wallis test and Mann-Whitney U test. In the symmetric distribution of continuous variables, to display the mean values and dispersion measures, we used the arithmetic mean and standard deviation, and their comparison was done with Student’s t-test. SPSS for Windows (version 17.0, SPSS Inc., Chicago, IL, USA) and Apache OpenOffice (version 3.3.0, The Apache Software Foundation, Wilmington, DE, USA) were used for statistical analyses. The values were considered statistically significant at p<0.05 (2-tailed).

## RESULTS

Infections were identified in 43 out of 240 (17.9%) hospitalised patients. In 27 (62.8%) cases of infection, the microbial agent was identified, while in 16 (37.2%) cases, no isolation was possible. The most common pathogen found was Pseudomonas aeruginosa. The frequency of gram-positive and gram-negative bacteria was almost the same. In 10 out of 27 cases, more than 1 microorganism was isolated (in 9 cases 2 and in 1 case 3 agents were isolated at the same time). In all cases, the identified microorganisms were bacteria, without any proven viral or fungal isolates. The frequencies of isolated agents are shown in Table 2.

The most common sites of infections were the urinary system in 16 cases (37.2%) and the blood in 7 cases (16.3%). In 4 cases more than 1 site of infection was found (urinary system and blood, as well as upper respiratory tract and blood, both in 2 cases). In 8/43 cases (18.6%), the site of infection was not identified. The determined sites of infection are summarised in Table 3. The average age in the group of patients with infections was 69 years and in those without infections was 65 years, which did not reach statistical significance (p=0.094).

The average disease duration in the cases without infection was 17.7 months and in the cases with infection was 33.6 months, which was statistically significant (p=0.004).

In 18 of 43 cases, infections occurred in patients who had previously suffered from infective complication, which means that the re-infection rate was 41.9%. Fatal outcome related to infection occurred in 4/43 cases (9.3%).

According to the type of therapy, the highest frequency of infections occurred in the group treated with bortezomib (14/28) and the lowest with VAD protocol (5/76), as can be seen in Table 4.

Our results established that the factors associated with more frequent infections were female sex (p=0.001), poor general condition (p<0.001), IIIB 3B (advanced) Durie-Salmon stage of disease (p=0.007), increased serum creatinine (p=0.036), neutropenia (p=0.009), high serum ferritin level (p=0.001), and the presence of catheters, especially urinary (p<0.001). There was no difference in infection occurrence related to the patient’s age (p=0.072), ISS score (p=0.532), or presence of immuneparesis (p=0.278).

## DISCUSSION

To date, there have been scant data regarding the incidence and characteristics of infections in hospitalised myeloma patients, especially those who were not treated with high-dose therapy and HSCT. According to our results, infections occurred in 17.9% cases of myeloma patients hospitalised in our ward during a 3-year period. The most frequently isolated bacteria were Pseudomonas aeruginosa, Escherichia coli, Staphylococcus epidermidis, and Enterococcus faecalis, and the numbers of gram-positive and gram-negative infections were almost equal, which is in concordance with the existing data [10,20]. These agents caused mainly urinary tract infections and bacteraemia. Contrary to recent reports that showed an increased incidence of fungal infections [12,13,14,15], none of these pathogens or fungal surrogate markers were detected in cases when fungal infection was part of a differential diagnosis [12,13,14,15]. This conflicting finding can be explained by the fact that our patients were not treated with high-dose therapy at the time of this study. Because of that, prolonged and severe neutropenia and mucositis, which are well-known risk factors for invasive fungal infections, occurred rarely in our group.

Traditionally, the frequency of infections is the highest during the first few months of induction therapy and in the later course of advanced disease [9,20,21,22,23,24,25,26]. On the contrary, patients who respond to therapy and those in the “plateau” phase are at low risk [24]. In our sample, the average disease duration in the cases without infections was significantly shorter than in the cases with infections, which implies that infections tend to occur in the later stage of relapsed or refractory disease. This result supports the idea that uncontrolled and long-lasting myeloma increases cumulative immunosuppression and represents an important infection risk factor. Moreover, these patients more often suffer from renal failure, neutropenia, bone fractures, neuropathy, and other established infection promoters. Our results suggest that these subsets of patients are also candidates for antimicrobial prophylaxis, just as the patients during the first months of induction therapy, which has been proposed by some other researchers [27,28].

The data regarding the mortality rate in myeloma patients who contract infective complications are quite limited. Considering the fact that a significant number of our patients suffered from progressive disease, poor performance status, and high incidence of bacteraemia, the mortality rate of 9.3% seems acceptable.

Anderson et al. reported a significant proportion of infections after the VAD regimen, which was probably related to dexamethasone-induced T-cell immunodeficiency [29]. Thalidomide is not heavily myelotoxic and the infection risk is not too high when it is used. However, the incidences of infections and pneumonia are higher in MPT than in MP groups of patients in some (but not all) clinical studies, which can be related to the immunomodulatory functions and indirect effects on deep vein thrombosis and peripheral neuropathy [30,31,32,33,34,35]. Bortezomib is also not significantly myelotoxic, but it mediates potent immunosuppressive effects on T cells, strongly connected with herpes zoster infection and other infections [36,37,38,39,40,41]. The infection rate in patients treated with bortezomib is variable, but Mateos et al. reported that 75% of patients treated with melphalan, prednisone, and bortezomib had at least 1 episode of infection during treatment [42]. According to our results, patients treated with the VAD regimen had the lowest infection incidence (6.6%), but, as already mentioned, VAD was used as the first-line therapy. The frequency of infections was higher with dexamethasone-monotherapy (12.7%) and thalidomide-based protocols (15%), both commonly used as second-line therapies. The highest rate of infections was in the “bortezomib-based protocols” group (50%). As explained above, bortezomib-based protocols were used in this study as salvage treatment for refractory/progressive disease in patients with severe cumulative malfunction of the immune system, which is thought to be the main reason for the highest infection rate in patients treated with bortezomib. On the other hand, no herpes zoster infection was noticed in our group at the time of study, probably because of the prophylactic use of acyclovir.

Among our cases, almost 80% of all infective complications were restricted to the female sex, which is quite an interesting result. On the other hand, some authors found a higher infection rate among men [24,43]. Hargreaves et al. suggested that cigarette smoking, which was more typical for men, was one of the possible explanations for this [24]. The reasons for more frequent infections in women, especially possible differences in the infection sites, and urinary catheter application among women and men should be analysed in a future study on a representative number of patients.

In our patients, the highest infection rate was observed in those with poor performance status, i.e. WHO/ECOG Groups 3 and 4. This finding was expected because this group of patients very frequently had other infection risk factors such as advanced age, progressive/uncontrolled disease, renal dysfunction, or different catheters.

No difference in infection rate was found between different ISS groups of patients, but advanced Durie-Salmon Stage IIIB 3B was significantly associated with more frequent infective complications. Contrary to some other studies [43,44], this result supports the idea that large tumour burden, especially when associated with renal failure, is an important infection risk factor in myeloma patients [43,44]. Like in other groups, a positive correlation between renal dysfunction and infections was found and these patients had a significantly higher rate of infective complications [10,43,45,46,47,48,49], which should be included in the estimation of infection risk [10,43,45,46,47,48,49].

In contradiction to other studies [10,43,45,46,47,48], the prognostic importance of serum polyclonal immunoglobulin deficiency as a risk factor for infection was not confirmed in our sample [10,43,45,46,47,48]. However, the present study evaluated immuneparesis only qualitatively, not quantitatively, and the number of patients without immuneparesis was low, which could have influenced and limited our results.

Our data confirmed the well-established fact that the degree of neutropenia in haematological diseases is related to the risk of infections [50,51]. The majority of our cases had no neutropenia. However, patients with neutrophil counts of less than 1x109/L had a higher infection rate. A previous randomised trial showed some benefits for severely neutropenic myeloma patients after HSCT treated with granulocyte colony-stimulating factor (G-CSF) [52]. Although the prophylactic use of G-CSF in neutropenic myeloma patients is supported on an everyday basis, the question of whether prophylactic use or addition of G-CSF to broad-spectrum antibiotics in non-transplanted myeloma patients really improved their outcome is still open and requires new clinical trials.

This study has shown that patients with high serum ferritin, and especially those with levels twice the norm (caused by an underlying disease, their treatment, blood transfusions, or the infection itself), had a greater infection incidence, which some authors have already suggested, particularly in patients treated with high-dose therapy [11,53]. This suggests that iron overload, and especially free iron, can cause immunosuppression, probably due to the damaging of polymorphonuclear and macrophage function, T cell development, and facilitation of the growth of microorganisms [54].

Indwelling central venous and urinary catheters are important devices in haematological disease management but catheter-bearing patients have impaired mucosal integrity and are at significant risk of infection complications [55,56,57]. In our group, patients with different catheters, especially urinary, had an increased infection rate in comparison to those without catheters. This result highlights the importance of our decisions and estimations of possible risk and benefit regarding the implantation of different catheters. The rate of re-infections among our patients was rather high, which implies that a previous infection represents another infection risk factor.

Over the last decades we have witnessed remarkable progress in the treatment of MM, which resulted in the prolongation of survival rates. However, these accomplishments have not been achieved only because of transplant procedures and potent novel drugs, but also because of better supportive therapy including anti-infective drugs.

According to our results, female sex, advanced clinical stage and WHO/ECOG performance status, neutropenia, increased levels of serum creatinine and ferritin, presence of catheters (especially urinary), and previous infections represent infection risk factors in patients with MM. Each myeloma patient, especially those with one or more of the mentioned risk factors, should be carefully and individually studied in order to decrease the incidence and severity of infective complications using different approaches.

## Figures and Tables

**Table 1:**
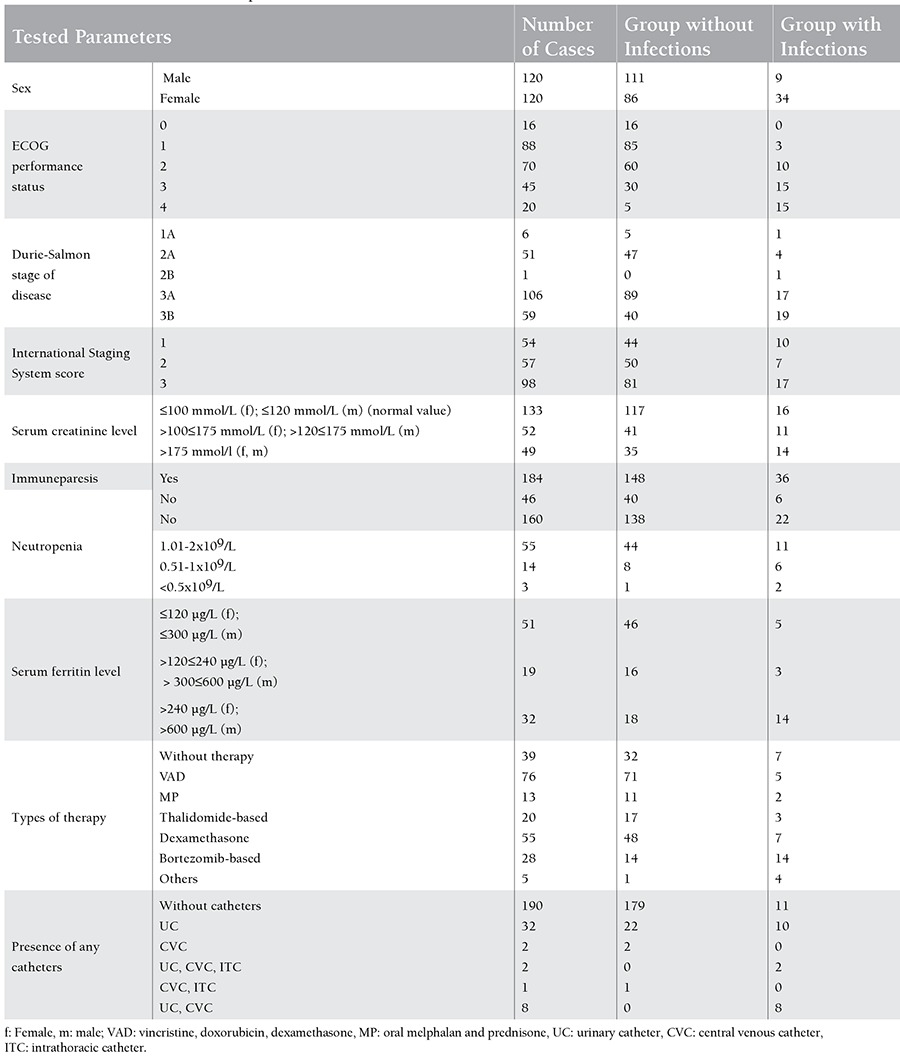
Characteristics of tested parameters.

**Table 2:**
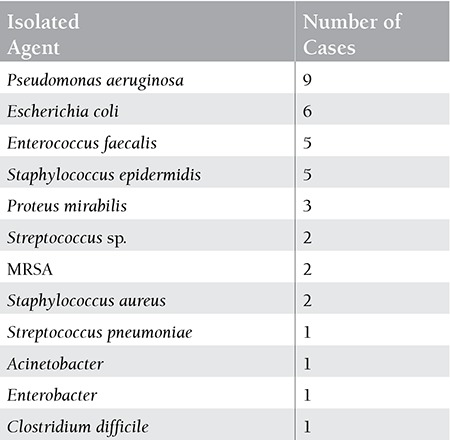
The frequencies of isolated agents.

**Table 3:**
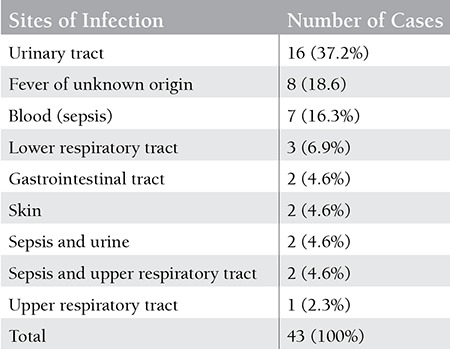
The determined sites of infection.

**Table 4:**
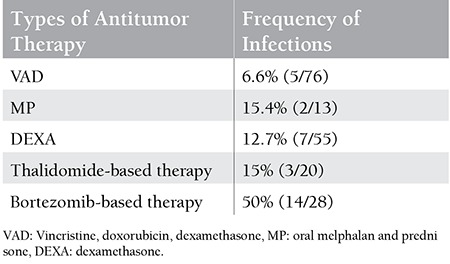
Frequency of infections based on type of antitumor therapy.
